# Temporal trends and global burden of urolithiasis: a comparative analysis of incidence, prevalence, mortality, and disability-adjusted life years in China and globally from 1990 to 2021

**DOI:** 10.3389/fepid.2025.1623575

**Published:** 2025-11-24

**Authors:** Chao Ma, Linlin Chen

**Affiliations:** 1 Department of Urology, The First Affiliated Hospital of Zhengzhou University, Zhengzhou, Henan, China; 2 Department of Cardiology, The First Affiliated Hospital of Zhengzhou University, Zhengzhou, Henan, China

**Keywords:** urolithiasis, age-standardized rates, incidence, prevalence, mortality, disability-adjusted life years (DALYs), healthcare disparities, sex-specific analysis

## Abstract

**Background:**

Urolithiasis significantly affects global health, contributing to substantial morbidity, healthcare costs, and reduced quality of life. Understanding temporal changes in the incidence, prevalence, mortality, and age-standardized disability-adjusted life year rates (ASDR) associated with urolithiasis is crucial for public health planning. However, few studies have systematically compared national and global trends, particularly in countries undergoing rapid healthcare transformation, such as China.

**Methods:**

Using data from the Global Burden of Disease database from 1990 to 2021, we assessed the age-standardized incidence rate (ASIR), prevalence rate (ASPR), mortality rate (ASMR), and ASDR associated with urolithiasis in China and globally. Joinpoint regression was used to identify trend changes, and sex-specific subgroup analyses were performed.

**Results:**

From 1990 to 2021, China showed substantial declines in all burden metrics: ASIR (−1.99%), ASPR (−1.99%), ASMR (−3.83%), and ASDR (−3.27%). Global declines were more modest: ASIR (−0.81%), ASPR (−0.81%), ASMR (−1.20%), and ASDR (−1.05%). Males consistently bore a higher burden.

**Conclusions:**

The burden of urolithiasis has declined markedly from 1990 to 2021, with China showing greater improvements than the global average. This divergence suggests that systemic health reforms and expanded coverage may have contributed to the observed trends. Comparative findings imply that promoting equitable access to prevention and early intervention could be beneficial, particularly in resource-limited settings.

## 1 Introduction

Urolithiasis, defined as the formation of calculi within the urinary tract, is a common cause of morbidity and is often associated with acute renal colic, hematuria, and recurrent urinary tract infections. If not properly managed, it can lead to serious complications, including obstructive uropathy, hydronephrosis, and impaired renal function (1, 2). In 2021, an estimated 106 million new cases of urolithiasis were recorded globally, with more than two-thirds occurring in men. Although the global age-standardized incidence rate (ASIR) declined from 1,450 per 100,000 in 2,000 to 1,240 per 100,000 in 2021—a 17.5% reduction after adjusting for demographic changes (2). The burden of urolithiasis remains substantial and varies considerably across geographical regions, age groups, and sexes, with consistently higher rates in males than in females (3, 4). Despite its widespread prevalence, comprehensive data exploring long-term global trends in the incidence, prevalence, mortality, and disability-adjusted life years (DALYs) of urolithiasis remain relatively limited. A clear understanding of these epidemiological patterns is crucial for informing and refining targeted public health strategies, particularly given the persistent regional disparities between high- and low- to middle-income countries (5).

Regional differences—especially comparisons between China and global averages—offer unique insights into the impact of demographic transitions, socioeconomic changes, and healthcare system reforms on disease burden. China represents an ideal setting for such a comparative study due to its rapid urbanization, significant population aging, extensive healthcare reforms, and evolving lifestyle patterns (6). Examining how China's experience aligns or diverges from global trends can inform best practices and provide valuable lessons for other countries seeking to mitigate urolithiasis burden. Previous research has identified various factors influencing urolithiasis trends, including demographic shifts, healthcare advancements, dietary modifications, and socioeconomic factors (7). For instance, rapid urbanization accompanied by increased consumption of processed foods and reduced fluid intake has elevated the prevalence of kidney stones in specific regions (8, 9). On the other hand, advances in healthcare access and preventive strategies have helped alleviate the burden in certain populations. Disparities in healthcare infrastructure, socioeconomic conditions, and diagnostic capacity continue to shape disease outcomes worldwide (10).

Although previous studies have significantly advanced our understanding of the urolithiasis burden, few have undertaken long-term, side-by-side comparisons between global patterns and national trajectories, such as those observed in China, using standardized GBD indicators like ASIR, ASPR, ASMR, and ASDR (2, 8–10). Even fewer have attempted to contextualize these trends within evolving national health policy environments. By conducting a 30-year comparative analysis based on harmonized GBD 2021 data, this study contributes a unified perspective on global and Chinese trends and explores how shifts in disease burden may relate to broader health system changes.

Therefore, key questions remain: What are the precise temporal trends in the burden of urolithiasis at both global and national levels, particularly in China? How have these trends evolved across different sexes and age groups? Have China's healthcare policies and preventive strategies led to measurable improvements compared to global benchmarks? To address these questions, this study utilizes data from the Global Burden of Disease (GBD) 1990–2021 dataset, focusing on age-standardized rates of incidence (ASIR), prevalence (ASPR), mortality (ASMR), and disability-adjusted life years (ASDR). Through comparative trend analysis, we aim to identify critical epidemiological patterns and generate policy-relevant insights to support future urolithiasis prevention and control efforts worldwide.

## 2 Materials and methods

### 2.1 Overview

This study aimed to comprehensively analyze temporal trends in the burden of urolithiasis globally and in China from 1990 to 2021. Using data from the GBD 2021 study, we evaluated changes in ASIR, ASPR, ASMR, and ASDR of urolithiasis. The trends were further examined according to demographic subgroups, including age and sex. A detailed comparison between global and China-specific data was conducted to highlight regional variations, focusing on differences potentially attributable to healthcare improvements and prevention strategies.

### 2.2 Data sources

The present analysis utilized publicly available estimates from the Global Burden of Disease (GBD) 2021 study, coordinated by the Institute for Health Metrics and Evaluation (IHME). The GBD compiles and integrates epidemiological information from a variety of sources—including population-based surveys, hospital discharge data, vital registration systems, scientific publications, and international health databases—to ensure comparability across countries and time.

For this study, data concerning urolithiasis incidence, prevalence, mortality, and disability-adjusted life years (DALYs) between 1990 and 2021 were extracted for both China and the global population. The estimates were standardized by age and sex to allow cross-population and temporal comparisons. Uncertainty intervals (95% UIs) were provided to quantify data variability and estimation precision.

The modeling framework employed in GBD 2021—such as the DisMod-MR 2.1 Bayesian meta-regression tool and the Cause-of-Death Ensemble Model (CODEm)—was used to derive consistent estimates even for years affected by healthcare disruptions, such as during the COVID-19 pandemic (2).

### 2.3 Measures of disease burden

The burden of urolithiasis was quantified using four core indicators defined by the Global Burden of Disease (GBD) framework: incidence, prevalence, mortality, and disability-adjusted life years (DALYs).

Incidence refers to the number of newly diagnosed urolithiasis cases occurring within a specific year.

Prevalence represents the total number of individuals affected by urolithiasis at a given point in time, including both newly and previously diagnosed cases.

Mortality corresponds to deaths directly attributed to urolithiasis, expressed as an age-standardized rate.

DALYs combine years of life lost due to premature mortality with years lived with disability, providing a comprehensive measure of the overall health loss associated with the disease.

Each indicator was age-standardized and expressed per 100,000 population to ensure valid comparisons between different regions and across time periods, in accordance with GBD methodological standards. These standardized rates enable consistent interpretation of disease trends and facilitate comparison with previous global and national estimates.

### 2.4 Statistical analysis and joinpoint regression

Temporal trends in ASIR, ASPR, ASMR, and ASDR from 1990 to 2021 were analyzed using joinpoint regression, a widely recognized method for identifying statistically significant changes in disease trends over specified periods. Joinpoint regression segments temporal data into distinct linear segments joined at points where significant shifts in trends occur (“joinpoints”). Annual percent change (APC) was computed for each segment, and the overall trend from 1990 to 2021 was summarized by calculating the average annual percentage change (AAPC). Statistically significant joinpoints indicate substantial epidemiological shifts, possibly attributable to public health interventions or changes in risk factors.

The joinpoint regression model was particularly suited to this study, as it enabled the identification of inflection points where the trajectories of urolithiasis burden in China diverged from or converged with global patterns. This approach allowed for a more dynamic understanding of epidemiological transitions and provided evidence of how national health policies and socioeconomic shifts may have influenced these trends differently across populations.

In this analysis, we employed the Joinpoint Regression Program software (version 5.2.0.0, April 2024; Statistical Research and Applications Branch, National Cancer Institute, United States of America). The number of joinpoints was determined automatically using the permutation test method, with significance set at *p* < 0.05. Confidence intervals (95% CIs) were provided to quantify the uncertainty in the APC and AAPC estimates, facilitating a transparent interpretation of temporal trends.

In addition, the comparative analysis between China and global estimates further highlights the methodological innovation of this work, as joinpoint regression was applied not only to detect overall trends but also to contrast the timing and magnitude of epidemiological shifts between different population contexts.

### 2.5 Additional analyses

Additional statistical analyses, including data extraction, preliminary data cleaning, visualization, and subgroup analyses by sex, were performed using R statistical software program (version 4.4.1). Significance tests were performed using a threshold of *p* < 0.05, indicating robust statistical evidence.

### 2.6 Data reliability and uncertainty intervals

Each GBD estimate is accompanied by a 95% uncertainty interval (UI) to capture both sampling and modeling uncertainty. These UIs, derived through Bayesian meta-regression implemented in DisMod-MR 2.1 and the CODEm framework, reflect variability across data sources and regional heterogeneity. Wider UIs typically indicate limited or heterogeneous data inputs, warranting cautious interpretation. The presentation of UIs follows standard GBD reporting practices to maintain transparency and reproducibility (10, 11).

### 2.7 Ethical considerations

Ethical approval was not required for this study because all analyses utilized publicly accessible, aggregated, and de-identified data provided by the GBD 2021 database. No individual-level data were used to ensure complete anonymity and confidentiality.

### 2.8 Reporting guidelines

The methodological approach used in this study strictly adhered to the GBD protocol and international reporting standards for epidemiological research. The results were transparently reported according to the recommendations for observational studies, with age-standardized rates and uncertainty intervals clearly presented. This ensured comparability with previous studies and facilitated the reproducibility of the analyses.

## 3 Results

### 3.1 Description of the burden of urolithiasis in China and worldwide

#### 3.1.1 Incidence of urolithiasis in China and worldwide

From 1990 to 2021, the ASIR of urolithiasis significantly declined in China from 1,793.16 to 964.7 per 100,000 people (AAPC: −1.99%, 95% CI: −2.03% to −1.94%). Conversely, the global ASIR decreased moderately from 1,602.48 to 1,242.84 per 100,000 (AAPC: −0.81%, 95% CI: −0.84% to −0.78%) (Table 1).

**Table 1:** Comparison of all-age cases and ASIR, ASPR, ASMR, and ASDR of urolithiasis in China and globally in 1990 and 2021, with corresponding AAPC and 95% CI.

Location	Measure	1990 All-ages cases *n* (95% Cl)	1990 Age-standardized rates per 100,000 people *n* (95% Cl)	2021 All-ages cases *n* (95% Cl)	2021 Age-standardized rates per 100,000 people *n* (95% Cl)	1990–2021 AAPC *n* (95% Cl)
	Incidence	18,072,865 (14,659,952–22,588,549)	1,793.16 (1,446.02–2,235.14)	19,141,120 (15,680,545–23,651,492)	964.7 (801.26–1,175.09)	−1.99 (−2.03 to −1.94)
China	Prevalence	680,491 (548,275–843,469)	67.76 (54.53–83.85)	726,452 (592,679–889,890)	36.45 (30.07–44.01)	−1.99 (−2.03 to −1.94)
	Deaths	3,350 (1,309–4,541)	0.51 (0.18–0.7)	2,859 (1,838–4,042)	0.15 (0.1–0.21)	−3.83 (−4.20 to −3.46)
	DALYs	141,008 (80,661–182,834)	15.7 (8.55–20.26)	111,364 (85,195–142,949)	5.63 (4.31–7.27)	−3.27 (−3.46 to −3.09)
Global	Incidence	73,115,604 (60,446,950–90,045,280)	1,602.48 (1,315.54–1,983.36)	105,983,780 (88,349,356–128,645,155)	1,242.84 (1,034.94–1,506.99)	−0.81 (−0.84 to −0.78)
	Prevalence	2,762,517 (2,266,583–3,375,342)	60.72 (49.44–74.41)	4,020,836 (3,363,932–4,816,302)	47.1 (39.41–56.31)	−0.81 (−0.84 to −0.78)
	Deaths	10,900 (7,351–13,193)	0.29 (0.2–0.35)	17,672 (13,932–21,241)	0.21 (0.17–0.25)	−1.20 (−1.40 to −0.99)
	DALYs	495,083 (379,478–610,119)	11.38 (8.72–13.99)	693,444 (567,765–850,490)	8.15 (6.68–9.99)	−1.05 (−1.22 to −0.89)

#### 3.1.2 Prevalence of urolithiasis in China and worldwide

Between 1990 and 2021, China experienced a notable reduction in ASPR of urolithiasis, decreasing from 67.76 to 36.45 per 100,000 (AAPC: −1.99%, 95% CI: −2.03% to −1.94%). Globally, the ASPR also decreased, though less substantially, from 60.72 to 47.10 per 100,000 (AAPC: −0.81%, 95% CI: −0.84% to −0.78%) (Table 1).

#### 3.1.3 Mortality of urolithiasis in China and worldwide

ASMR related to urolithiasis showed a substantial decrease in China from 0.51 to 0.15 per 100,000 population (AAPC: −3.83%, 95% CI: −4.20% to −3.46%) between 1990 and 2021. Globally, the ASMR experienced a slower decline, decreasing from 0.29 to 0.21 per 100,000 (AAPC: −1.20%, 95% CI: −1.40% to −0.99%) (Table 1).

#### 3.1.4 Disability-adjusted life year rates of urolithiasis in China and worldwide

The ASDR due to urolithiasis in China significantly dropped from 15.70 to 5.63 per 100,000 (AAPC: −3.27%, 95% CI: −3.46% to −3.09%) during 1990–2021. Globally, the ASDR reduced moderately from 11.38 to 8.15 per 100,000 (AAPC: −1.05%, 95% CI: −1.22% to −0.89%) (Table 1).

### 3.2 Joinpoint regression analysis of urolithiasis burden in China and worldwide

#### 3.2.1 ASIR

**Figure 1:**
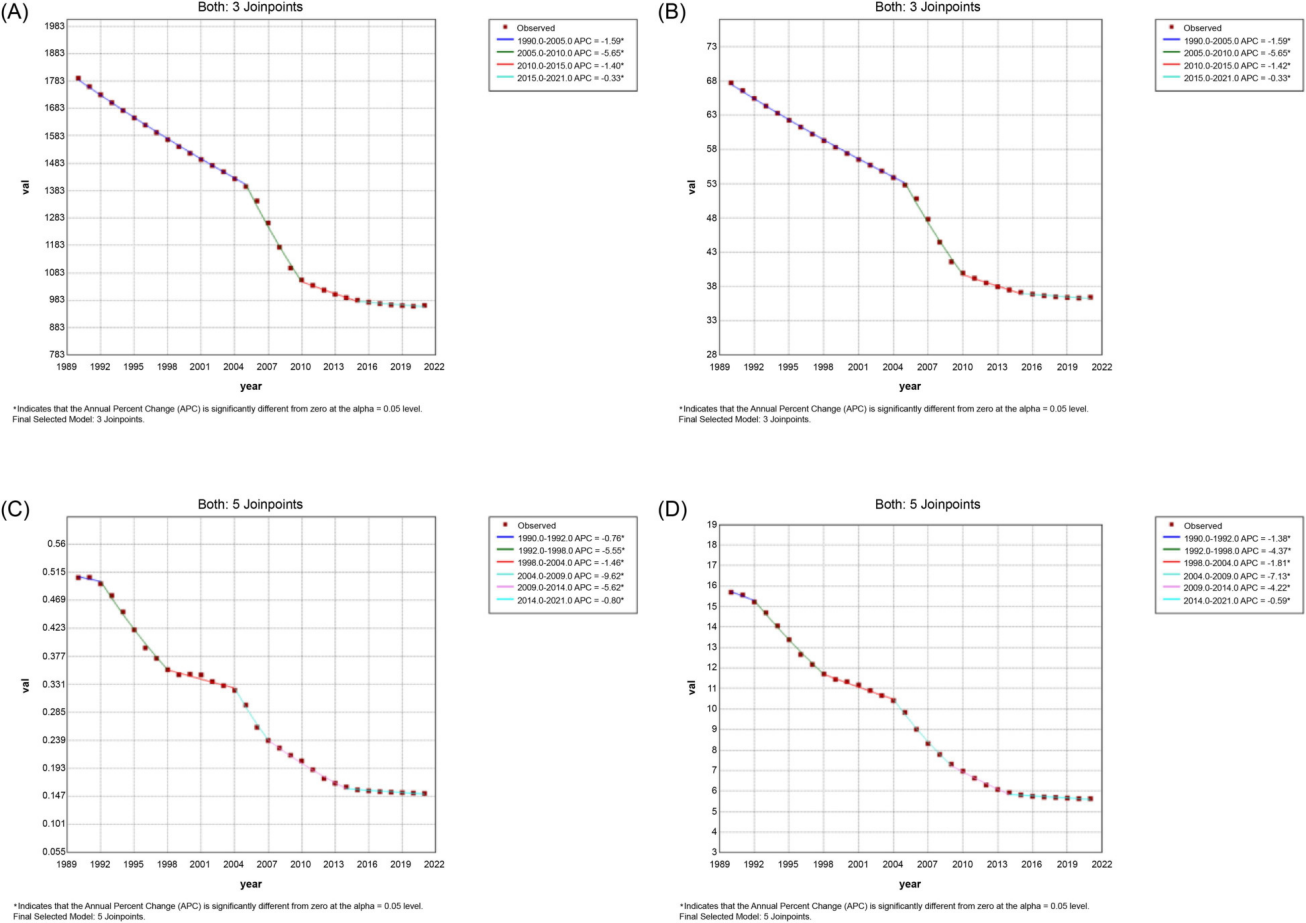
Joinpoint regression analysis of the age-standardized rates of urolithiasis in China, 1990–2021. Panels show temporal trends in **(A)** age-standardized incidence rate, **(B)** age-standardized prevalence rate, **(C)** age-standardized mortality rate, and **(D)** age-standardized disability-adjusted life year rate.

**Figure 2:**
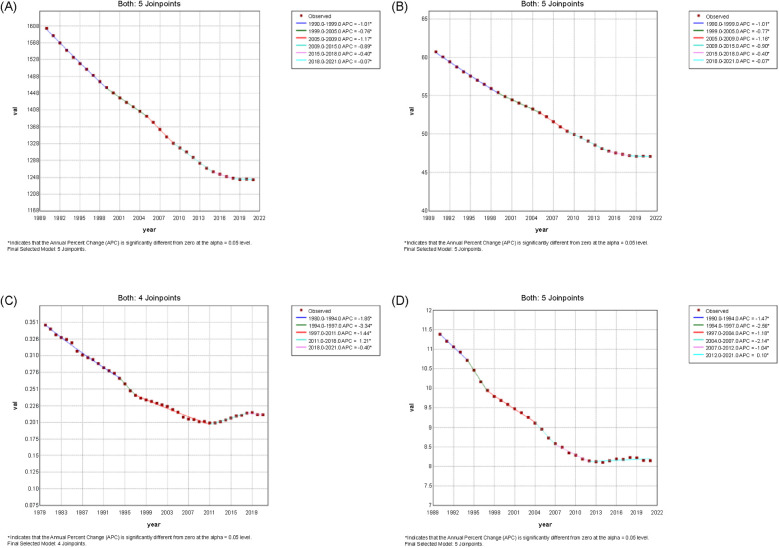
Joinpoint regression analysis of the age-standardized rates of urolithiasis globally, 1990–2021. Panels display trends in **(A)** age-standardized incidence rate, **(B)** age-standardized prevalence rate, **(C)** age-standardized mortality rate, and **(D)** age-standardized disability-adjusted life-year rate worldwide.

Figures 1A, 2A show the Joinpoint regression analysis of ASIR trends in China and globally from 1990 to 2021. In China, the most pronounced decline occurred between 2005 and 2010 (APC: −5.65%, 95% CI: −5.83% to −5.47%). The global ASIR exhibited a gradual reduction, with notable declines during 1990–1999 (APC: −1.01%, 95% CI: −1.04% to −0.99%) and 2005–2009 (APC: −1.17%, 95% CI: −1.30% to −1.04%).

#### 3.2.2 ASPR

Figures 1B, 2B display the Joinpoint regression analysis of ASPR trends. In China, ASPR declined between 2005 and 2010 (APC: −5.65%, 95% CI: −5.83% to −5.47%). The global ASPR followed a similar downward trend.

#### 3.2.3 ASMR

Figures 1C, 2C present ASMR trends. In China, a decline was observed between 2004 and 2007 (APC: −9.62%, 95% CI: −12.12% to −7.06%). Global ASMR also decreased, with shifts occurring during 1994–1997 (APC: −3.34%, 95% CI: −5.74% to −0.88%).

#### 3.2.4 ASDR

Figures 1D, 2D illustrate ASDR trends. In China, ASDR declined between 2004 and 2009 (APC: −7.13%, 95% CI: −7.57% to −6.68%). The global ASDR followed a similar pattern, with a steady decline over the study period.

#### 3.2.5 Trend parallelism tests

Trend parallelism tests revealed that the temporal trajectories of ASIR, ASPR, ASMR, and ASDR for urolithiasis in China significantly differed from those at the global level (all *p*-values < 0.001). These findings confirm that the decline patterns in China were not parallel to the global averages, suggesting substantial heterogeneity in epidemiological dynamics (Supplementary Table S1 and Figures S1–S4).

### 3.3 Comparison of age-standardized rates of urolithiasis between China and globally, 1990–2021

**Figure 3:**
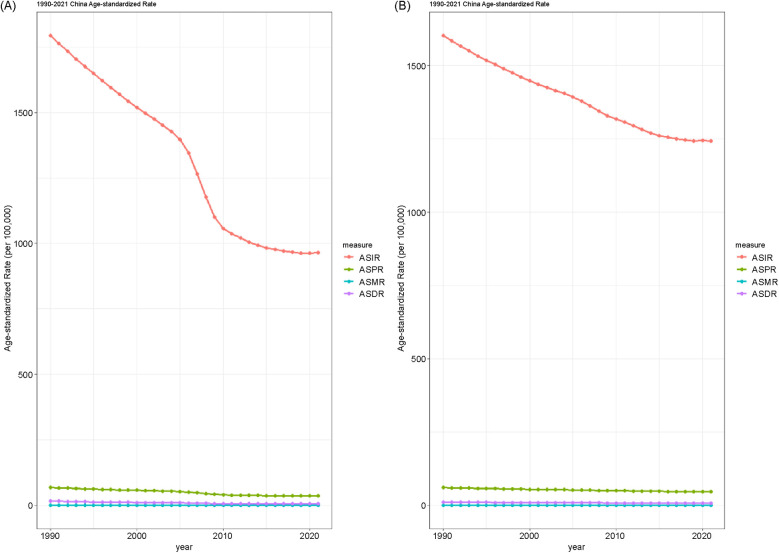
Illustrates the trends in age-standardized rates of urolithiasis from 1990 to 2021, comparing China **(A)** and global patterns **(B)** across four metrics: incidence, prevalence, mortality, and disability-adjusted life years.

Figure 3 illustrates the trends in age-standardized rates of urolithiasis from 1990 to 2021, comparing Chinese (Figure 3A) and global patterns (Figure 3B) across four metrics: incidence, prevalence, mortality, and DALYs.

#### 3.3.1 China

The ASIR declined during the study period, with a more apparent decrease in the early 2000s. The ASPR also showed a downward trend, though it was more gradual. The ASMR decreased steadily throughout the study period. The ASDR showed a continuous but moderate decline (Figure 3A).

#### 3.3.2 Global

The global ASIR decreased at a slower pace than in China, with a relatively stable trend after the early 2000s. The ASPR showed only slight changes over time, with minimal fluctuations. The ASMR declined gradually, with a consistent pattern across the years. The ASDR also followed a slow downward trend, with no abrupt shifts observed (Figure 3B).

### 3.4 Age-specific trends in urolithiasis burden in China, 1990–2021

**Figure 4:**
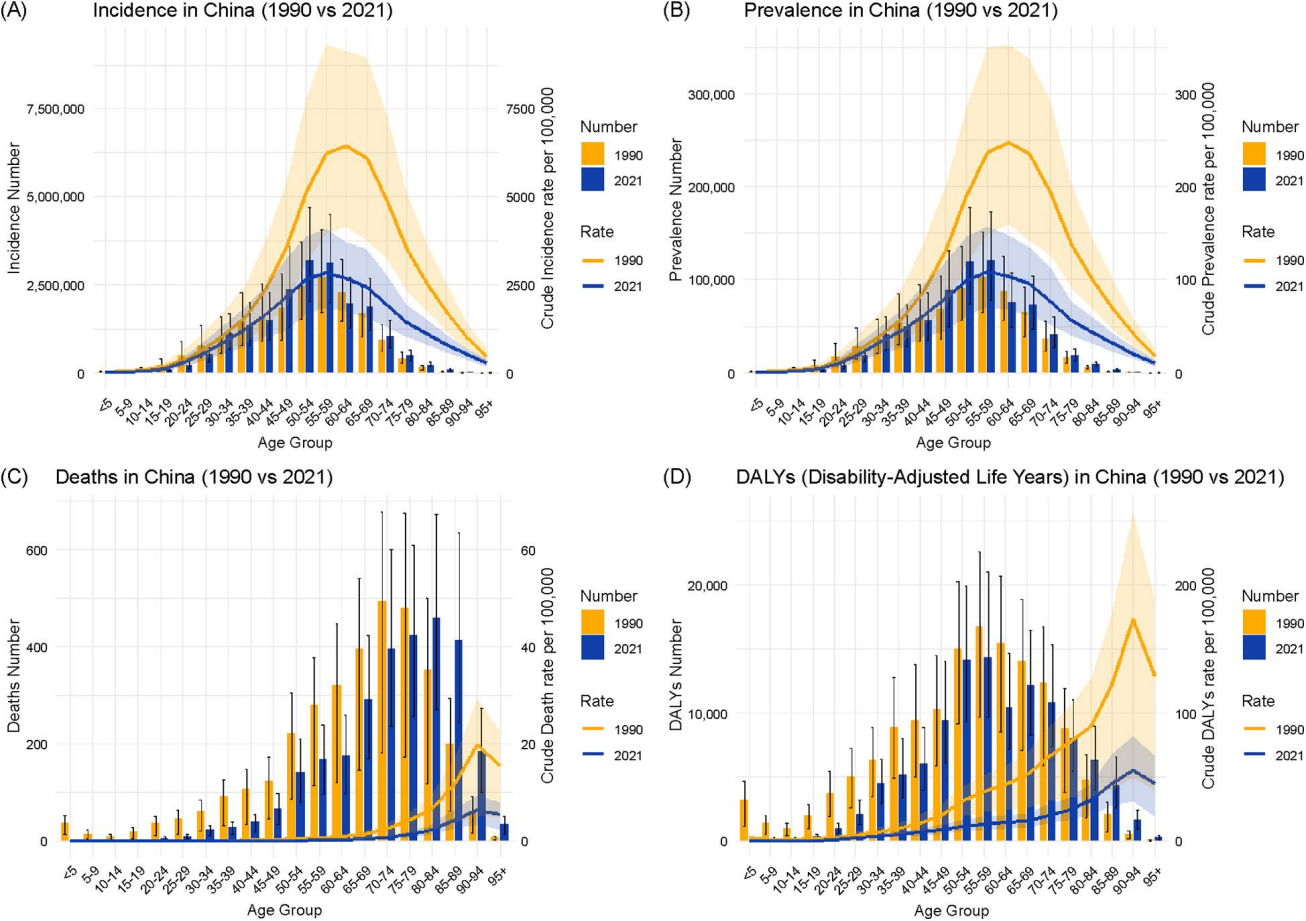
Illustrates the age-specific trends in the urolithiasis burden in China from 1990 to 2021, as depicted by the incidence **(A)**, prevalence **(B)**, deaths **(C)**, and disability-adjusted life years **(D).**

Figure 4 presents the age-specific trends in the incidence, prevalence, mortality, and DALYs of urolithiasis in China for 1990 and 2021. The figure includes both the number of cases (bars) and rates per 100,000 people (lines) to illustrate temporal changes across different age groups.

Incidence (Figure 4A): The highest incidence was observed in the 50–54 age group in both 1990 and 2021. The incidence per 100,000 people showed an overall decrease across all age groups, with a more pronounced reduction in the younger population.

Prevalence (Figure 4B): The highest prevalence was recorded in the 55–59 age group in 1990 and shifted to the 50–54 age group in 2021. The prevalence rate per 100,000 people was lower in 2021 than that in 1990 across most age groups.

Mortality (Figure 4C): The highest number of deaths occurred in the 75–79 age group in 1990 and in the 80–84 age group in 2021. The mortality rate per 100,000 people declined across all age groups, with a substantial reduction among older adults.

DALYs (Figure 4D): The greatest disease burden, measured by DALYs, was observed in the 60–64 age group in 1990 and in the 55–59 age group in 2021. A decline in DALYs was evident across all age groups, particularly in individuals aged 40 and above.

### 3.5 Age-specific trends in urolithiasis burden globally, 1990–2021

**Figure 5:**
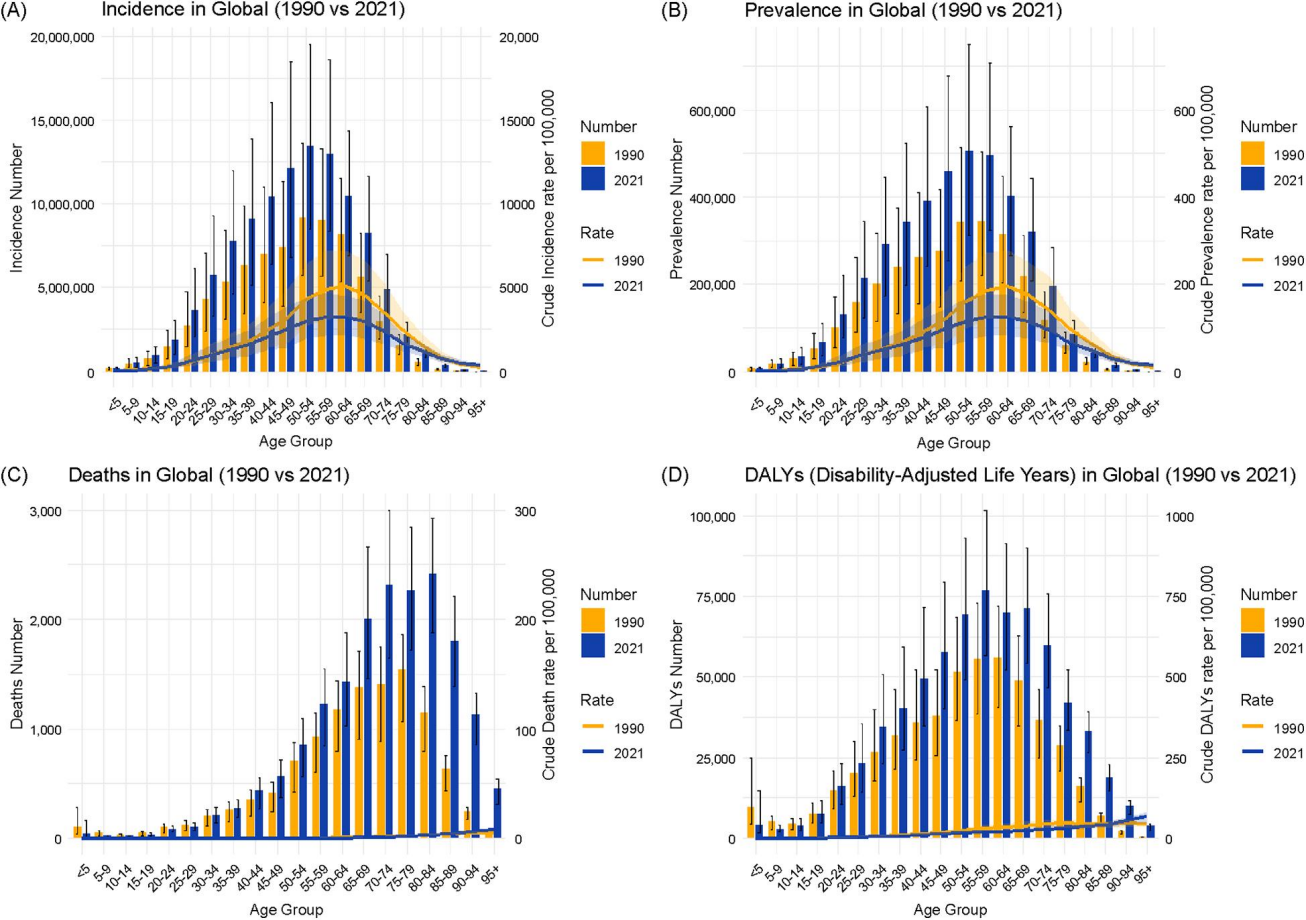
Illustrates age-specific trends in the global burden of urolithiasis from 1990 to 2021, including incidence **(A)**, prevalence **(B)**, deaths **(C)**, and disability-adjusted life years **(D)**.

Figure 5 presents age-specific trends in the incidence, prevalence, mortality, and DALYs of urolithiasis globally for 1990 and 2021. The figure includes both the number of cases (bars) and rates per 100,000 people (lines) to illustrate temporal changes across different age groups.

Incidence (Figure 5A): The highest incidence was observed in the 50–54 age group in both 1990 and 2021, which is consistent with the trend in China. However, the reduction in the incidence rate per 100,000 people is less pronounced globally than it is in China.

Prevalence (Figure 5B): The highest prevalence was recorded in the 55–59 age group in 1990 and in the 50–54 age group in 2021, similar to that in China. The global prevalence rate per 100,000 individuals declined slowly across most age groups.

Mortality (Figure 5C): The highest number of deaths occurred in the 75–79 age group in 1990 and in the 80–84 age group in 2021, mirroring the trend in China. However, the global mortality rate per 100,000 people declined at a slower rate than that in China.

DALYs (Figure 5D): The greatest disease burden, measured by DALYs, was in the 60–64 age group in 1990 and in the 55–59 age group in 2021. While DALYs declined globally, the reduction was more gradual than that in China.

### 3.6 Age and sex distribution of urolithiasis burden in China (1990 vs. 2021)

**Figure 6:**
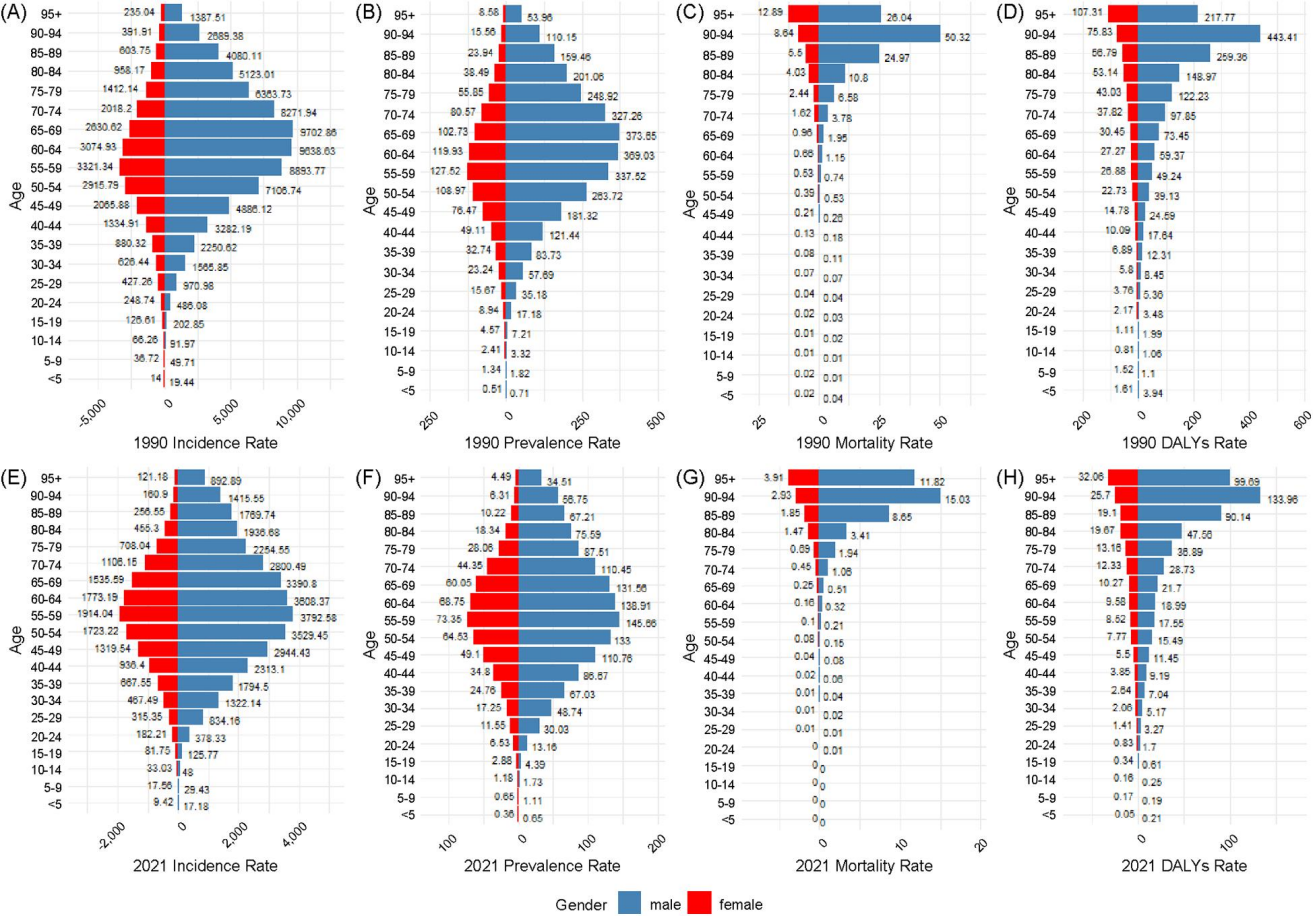
Depicts the age- and sex-specific distribution of the urolithiasis burden in China for 1990 and 2021, including incidence **(A,E)**, prevalence **(B,F)**, deaths **(C,G)**, and disability-adjusted life years **(D,H)**.

Figure 6 presents the age- and sex-specific distribution of the urolithiasis burden in China for 1990 and 2021, including incidence (6A, 6E), prevalence (6B, 6F), deaths (6C, 6G), and DALYs (6D, 6H). Each panel illustrates trends across different age groups.

#### 3.6.1 Incidence rate

In both 1990 and 2021, the incidence rate increased with age, peaking in the 60–64 age group in 1990 and shifting to the 55–59 age group in 2021. Across all age groups, males had consistently higher incidence rates than females. The male-to-female ratio was highest in the 25–29 age group (2.65 in 2021). Compared to 1990, the overall incidence rate declined in most age groups in 2021, with a greater reduction observed in younger individuals (<40 years) (Figures 6A,E).

#### 3.6.2 Prevalence rate

The prevalence rate peaked in the 60–64 age group in 1990 and shifted to the 55–59 age group in 2021. The male prevalence rate remained consistently higher than that of females across all age groups, with the largest male-to-female difference observed in the 25–29 age group (2.60 in 2021). Overall prevalence rates declined across most age groups, with smaller reductions observed in older age groups (≥60 years) (Figures 6B,F).

#### 3.6.3 Mortality rate

The highest mortality rate was observed in the 90–94 age group in both 1990 and 2021, with no significant shift over time. A notable sex disparity was observed in mortality rates across all age groups. The most pronounced difference was in the 5-year-old and younger age groups, where the male mortality rate was 7.52 times higher than that of females. The mortality rate declined across most age groups; however, the reduction was less pronounced in older populations (Figures 6C,G).

#### 3.6.4 DALYs rate

The DALYs rate was the highest in the 90–94 age group in both 1990 and 2021, with no significant shift over time. Males had consistently higher DALYs rates than females across all age groups, with the most pronounced sex disparity in the 25–29 age group (2.33 in 2021). A decline in DALYs rates was observed in most age groups, particularly among individuals aged 40 years. (Figures 6D,H).

### 3.7 Age and sex distribution of urolithiasis burden globally (1990 vs. 2021)

**Figure 7:**
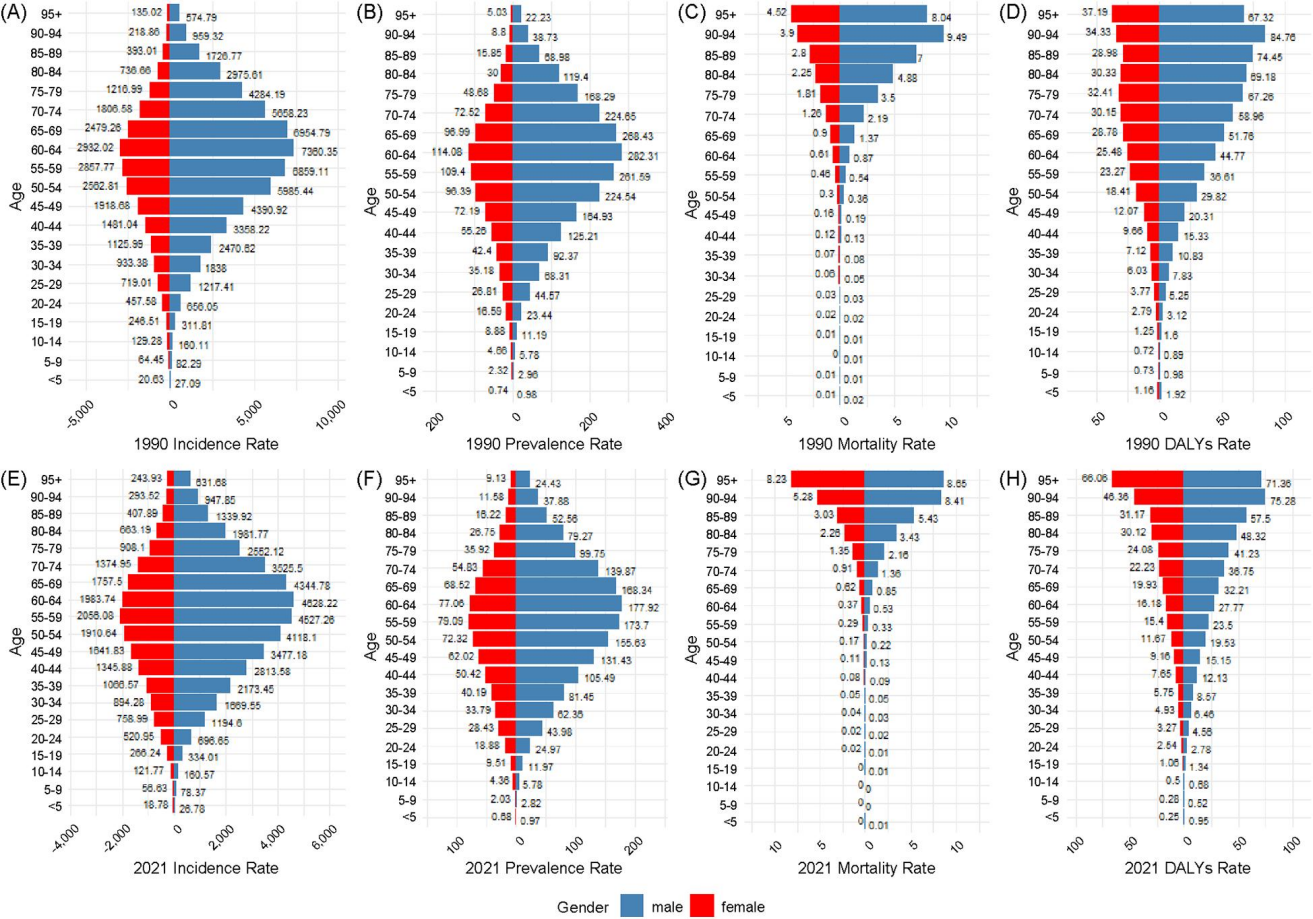
Illustrates the global age- and sex-specific distribution of the urolithiasis burden in 1990 and 2021, depicted as bilateral bar plots for incidence **(A,E)**, prevalence **(B,F)**, deaths **(C,G)**, and disability-adjusted life years **(D,H)**.

Figure 7 presents the age- and sex-specific global distribution of the urolithiasis burden for 1990 and 2021, including the incidence (7A, 7E), prevalence (7B, 7F), deaths (7C, 7G), and DALYs (7D, 7H). Each panel illustrates trends across different age groups.

#### 3.7.1 Incidence rate

In both 1990 and 2021, the incidence rate increased with age, peaking in the 60–64 age group. Across all age groups, males had consistently higher incidence rates than females. The male-to-female ratio was highest in the 85–89 age group (3.29 in 2021). Compared to 1990, the overall incidence rate declined in most age groups in 2021, with a greater reduction observed in younger individuals (<40 years) (Figures 7A,E).

#### 3.7.2 Prevalence rate

The prevalence rate peaked in the 60–64 age group in both 1990 and 2021. The male prevalence rate remained consistently higher than that of females across all age groups, with the largest male-to-female difference observed in the 90–94 age group (3.27 in 2021). Overall prevalence rates declined across most age groups, with smaller reductions observed in older age groups (≥60 years) (Figures 7B,F).

#### 3.7.3 Mortality rate

The highest mortality rate was observed in the 90–94 age group in 1990 and shifted to the ≥95-year age group by 2021. A notable sex disparity was observed in mortality rates across all age groups, with the highest male-to-female mortality ratio in the <5-year age group (4.38 in 2021). The mortality rate declined across most age groups, with smaller reductions observed in older populations (Figures 7C,G).

#### 3.7.4 DALYs rate

The DALYs rate was highest in the 90–94 age group in 1990 and shifted to the ≥95-year age group by 2021. Males had consistently higher DALY rates than females across all age groups, with the most pronounced sex disparity in the <5-year age group (3.76 in 2021). Most age groups showed a decline in DALY rates from 1990 to 2021, with a greater reduction observed in individuals aged 40 years and older (Figures 7D,H).

### 3.8 Sex-stratified temporal trends in urolithiasis burden in China (1990–2021)

**Figure 8:**
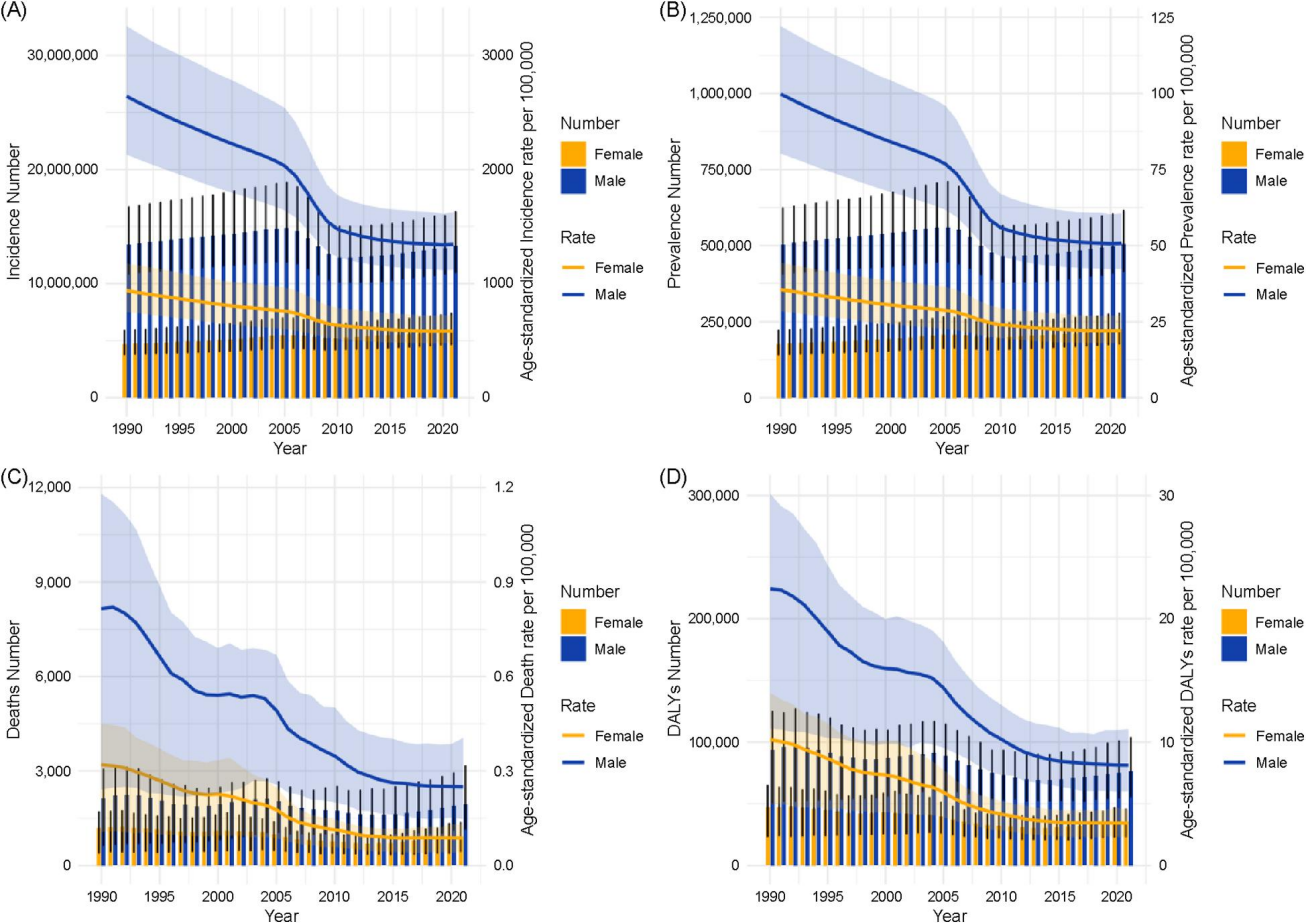
Shows dual-axis trends in the urolithiasis burden in China from 1990 to 2021, stratified by sex, including incidence **(A)**, prevalence **(B)**, deaths **(C)**, and disability-adjusted life years **(D)**.

Figure 8 shows the trends in the urolithiasis burden in China from 1990 to 2021, including incidence (8A), prevalence (8B), deaths (8C), and DALYs (8D). Each panel includes both the absolute number (bars) and age-standardized rate per 100,000 (lines), stratified by sex.

#### 3.8.1 Incidence

The total number of incident cases steadily increased from 1990 to 2021 for both sexes, with consistently higher counts in males. The ASIR declined over the same period, particularly in the early 2000s, with a more notable decrease observed in males than in females (Figure 8A).

#### 3.8.2 Prevalence

The number of prevalent cases gradually increased in both sexes. The ASPR showed a general decreasing trend, with a more substantial decline in males compared to females (Figure 8B).

#### 3.8.3 Deaths

The number of deaths from urolithiasis decreased slightly from 1990 to 2021, with males exhibiting consistently higher mortality counts. The ASMR declined in both sexes, with males experiencing a more substantial decrease (Figure 8C).

#### 3.8.4 DALYs

The number of DALYs followed a downward trend for both sexes, with higher absolute values for males. The ASDR consistently declined from 1990 to 2021 in both males and females (Figure 8D).

### 3.9 Sex-stratified temporal trends in global urolithiasis burden (1990–2021)

**Figure 9:**
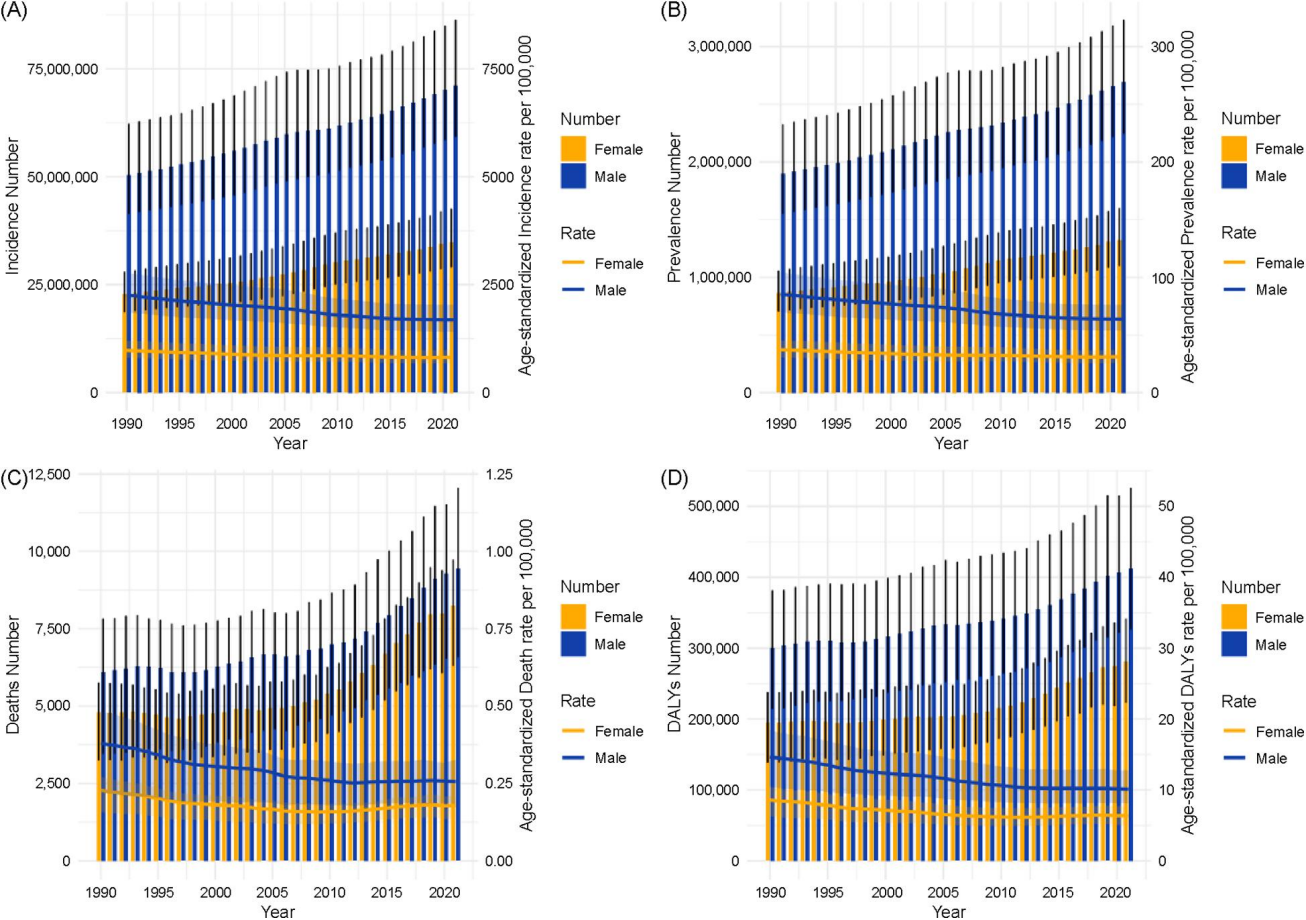
Shows dual-axis trends in the global urolithiasis burden from 1990 to 2021, stratified by sex, including incidence **(A)**, prevalence **(B)**, deaths **(C)**, and disability-adjusted life years **(D)**.

Figure 9 shows the global trends in the urolithiasis burden from 1990 to 2021, including incidence (9A), prevalence (9B), deaths (9C), and DALYs (9D). Each panel presents both the absolute number of cases (bars) and age-standardized rates per 100,000 people (lines) stratified by sex.

#### 3.9.1 Incidence

The total number of incident cases gradually increased over the study period in both sexes, with higher counts in males than in females. The ASIR showed a slight downward trend between 1990 and 2021 for both sexes (Figure 9A).

#### 3.9.2 Prevalence

The number of prevalent cases has steadily increased in both sexes. The ASPR remained relatively stable in females, whereas a modest decline was observed in males (Figure 9B).

#### 3.9.3 Deaths

The number of deaths showed minor fluctuations but remained relatively stable from 1990 to 2021. The ASMR declined slightly in both sexes, with rates consistently higher in males than in females (Figure 9C).

#### 3.9.4 DALYs

The number of DALYs declined modestly in males and remained relatively constant in females. The ASDR decreased gradually over the study period in both sexes, with a slightly more notable decline in males (Figure 9D).

## 4 Discussion

A key strength of this study lies in its comparative approach to analyzing the urolithiasis burden in both China and the global population. China was selected due to its rapid healthcare transformation over the past three decades, offering a valuable model for understanding how systemic reforms and public health strategies may influence disease burden (6, 12). Comparing China's progress with global trajectories allows for a clearer assessment of disparities in healthcare infrastructure, prevention efforts, and equity of access, providing insights relevant to diverse socioeconomic contexts (1, 2).

Furthermore, this study is among the few to apply joinpoint regression in a comparative cross-national context, enabling the identification of specific turning points where China's trends diverged from global averages. This analytical approach provided a dynamic means of detecting temporal shifts and quantifying how policy-driven transitions in China corresponded to broader global epidemiological changes.

Our analysis of Global Burden of Disease (GBD) 1990–2021 data revealed an overall decline in the burden of urolithiasis, with China demonstrating more pronounced reductions in age-standardized incidence, prevalence, mortality, and disability rates compared to global averages (2, 13). These improvements are likely attributable to a combination of expanded healthcare access, enhanced diagnostic capacity, public health campaigns promoting hydration and dietary modification, and increased rural service coverage (5, 14–15). Among these contributing factors, health system reforms, particularly the launch of the New Rural Cooperative Medical Scheme (NRCMS) in 2003, warrant special attention for their potential role in improving disease prevention and management (16, 17).

The NRCMS rapidly expanded to cover over 95% of China's rural population by 2010, achieving near-universal health insurance by 2011 (16). This expansion significantly improved access to basic medical services, enabling earlier detection and management of a wide range of health conditions, including those directly related to urolithiasis (12, 14). Between 2004 and 2010, a notable decline in China's ASIR and ASPR was observed, temporally coinciding with the rollout of NRCMS (Supplementary Table S2). While causality cannot be definitively established, this alignment suggests that improving equitable access to healthcare in underserved populations may have played a role in the observed reductions in urolithiasis burden (6, 18). Through the joinpoint regression analysis, we were able to statistically validate these inflection periods, supporting the notion that the most significant reductions in China's burden coincided with key health policy reforms. Such precision in trend segmentation provides stronger evidence than simple linear analyses, underscoring the methodological advantage of the joinpoint approach in public health time-series research. Beyond the overall reductions, the parallelism analyses further highlighted that China's trends diverged significantly from global patterns across all four indicators. This statistical evidence strengthens the argument that structural factors unique to China—such as the rapid rollout of the New Rural Cooperative Medical Scheme (NRCMS) and subsequent integration of urban and rural insurance schemes—may have accelerated the decline in urolithiasis burden compared with the global average. In particular, the more pronounced declines in incidence and prevalence between 2004 and 2010 temporally coincided with the expansion of NRCMS coverage, supporting the notion that equitable access to primary care and early diagnosis can alter disease trajectories at the population level.

In contrast, the global decline in urolithiasis burden was more modest. Many low- and middle-income countries continue to face persistent challenges, including limited diagnostic infrastructure, insufficient treatment capacity, and a lack of coordinated preventive services (5, 19). In addition, the absence of effective health system responses to rising rates of obesity and metabolic disorders may have further hindered progress in reducing the disease burden in these regions (20–22).

An important nuance lies in the observed discrepancy between declining age-standardized rates and increasing absolute numbers of cases or deaths. This pattern is likely explained by demographic changes, including population growth and aging (23). As life expectancy increases, the number of individuals at risk also rises, even if per capita rates fall. Additionally, improved imaging technologies and health information systems may have led to higher detection rates, complicating the interpretation of true incidence trends (13, 14).

Sex- and age-specific patterns further demonstrated that the urolithiasis burden remains disproportionately high among males and older adults. Tailored prevention and management strategies that account for age- and sex-related differences are particularly important for effectively addressing the needs of these vulnerable populations (15).

Technological advancements such as minimally invasive stone removal and improved imaging have contributed to declining disease burden in high-income countries, but these innovations are not uniformly accessible worldwide (4, 10). Notably, while advanced diagnostics can improve disease management, they may also lead to apparent increases in incidence due to greater case identification (13). Therefore, interpretation of time trends must consider the evolving landscape of diagnostic capacity (2, 11).

Despite the strengths of this study, including the use of standardized GBD data, age-adjusted metrics, and robust joinpoint regression, several limitations warrant discussion. First, GBD estimates are derived from statistical modeling techniques, and the accuracy of outputs depends on the quality of input data, particularly in low-resource regions (2, 23). Second, the ecological study design precludes causal inference and limits assessment of individual-level risk factors (3, 7). Third, the broader impacts of urolithiasis, such as financial hardship, lost productivity, and mental health stressors, were not captured in this analysis (24, 25).

Lastly, while the global comparator represents a heterogeneous reference group, we intentionally used it to highlight how one large country, China, responded to historically high disease burden through policy-driven reforms (6, 13). Future studies may consider cross-national comparisons between China and countries with similar healthcare or socioeconomic profiles to further explore the policy implications observed in this study.

Taken together, the application of joinpoint regression combined with a China–global comparative framework provides a novel methodological and analytical contribution to the study of disease burden trends. This dual approach enables more precise detection of policy-relevant shifts and fosters a deeper understanding of how national reforms interact with global health trajectories.

Beyond China, this experience underscores the potential of equity-oriented health reforms to reduce noncommunicable disease burdens in other low- and middle-income settings (5, 17, 18). In particular, expanding financial protection and improving access to primary care services in underserved populations may serve as effective strategies for mitigating the burden of conditions such as urolithiasis (18, 26).

To support future investigations into the policy–disease nexus, we compiled a chronological summary of major national health reforms in China from 1990 to 2021 (Supplementary Table S3). This reference may aid comparative analyses or inspire similar evaluations in other settings undergoing systemic health transitions.

## 5 Conclusions

This study leveraged data from the Global Burden of Disease (GBD) 1990–2021 to assess temporal trends in the burden of urolithiasis, focusing on age-standardized incidence, prevalence, mortality, and DALY rates in China and globally. The results revealed a substantial decline in China's urolithiasis burden, in contrast to more modest global improvements. These differences likely reflect variations in healthcare infrastructure, access to care, and implementation of preventive strategies. Notably, despite declining age-standardized rates, the absolute number of cases and deaths increased in some regions, likely due to population growth, aging, and enhanced diagnostic capabilities.

Addressing the global burden of urolithiasis requires sustained efforts to strengthen health systems, expand preventive measures, and improve data quality and comparability. Future research should investigate causal pathways and evaluate the effectiveness of targeted interventions across different socioeconomic and healthcare settings.

## Data Availability

Publicly available datasets were analyzed in this study. This data can be found here: https://ghdx.healthdata.org/gbd-2021. Repository Name: Global Health Data Exchange (GHDx). Accession Number: Not applicable; GBD 2021 datasets are openly accessible without the need for accession numbers.
