# Supplementary material for: Temporal trends and global burden of urolithiasis: a comparative analysis of incidence, prevalence, mortality, and disability-adjusted life years in China and globally from 1990 to 2021
Source: Front Epidemiol. 2025 Nov 24;5:1623575. doi: 10.3389/fepid.2025.1623575 (PMC12682779; doi:10.3389/fepid.2025.1623575)
Supplement: Supplementary file 1 [file Table1.docx]

# Supplementary Table S1. Results of Parallelism Tests between China and Global ASRs (1990–2021)

| Indicator | P-value | Conclusion |
| --- | --- | --- |
| ASIR | 0.000222 | Rejected Parallelism |
| ASPR | 0.000222 | Rejected Parallelism |
| ASMR | 0.000222 | Rejected Parallelism |
| ASDR | 0.000222 | Rejected Parallelism |

**Supplementary Figure S1. Trend Parallelism Test for ASIR (China vs. Global, 1990–2021)**


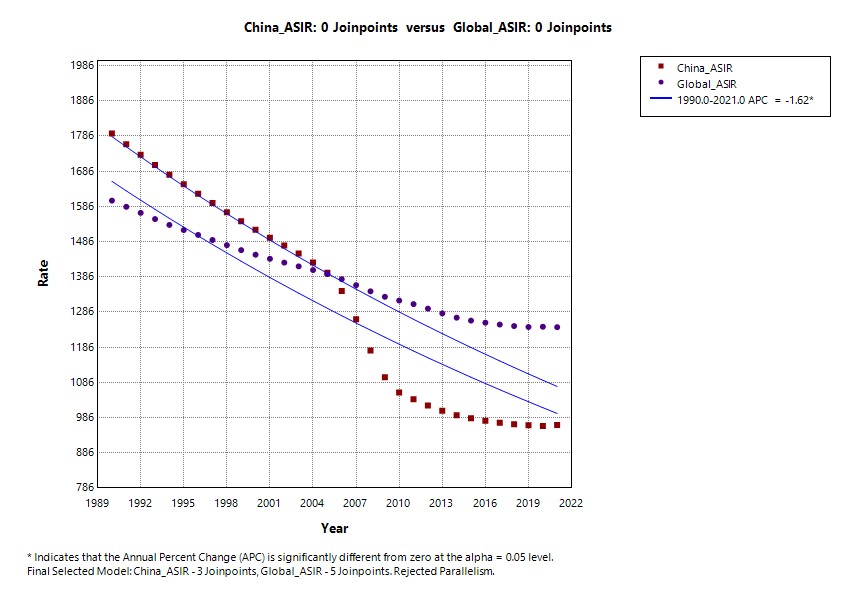


## **Supplementary Figure S2. Trend Parallelism Test for ASPR (China vs. Global, 1990–2021)**


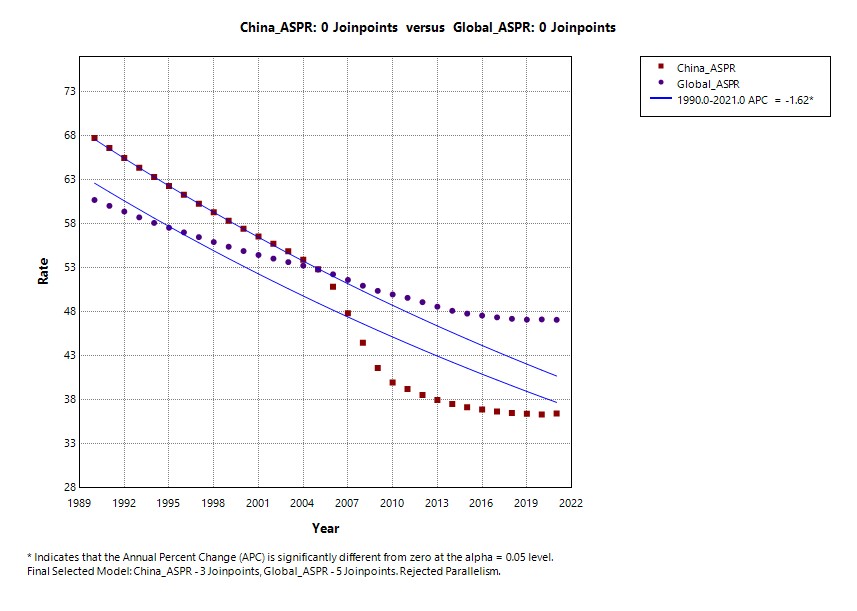


## **Supplementary Figure S3. Trend Parallelism Test for ASMR (China vs. Global, 1990–2021)**


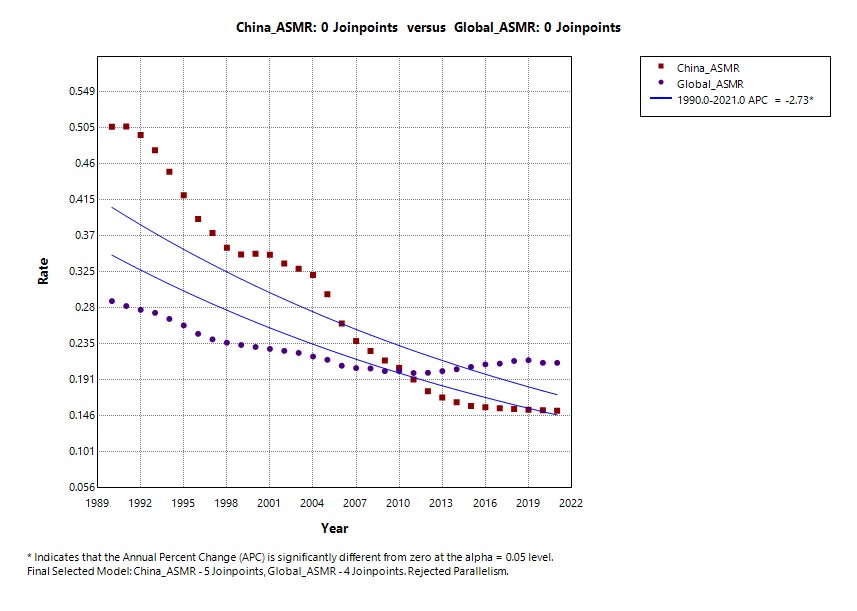


## **Supplementary Figure S4. Trend Parallelism Test for ASDR (China vs. Global, 1990–2021)**


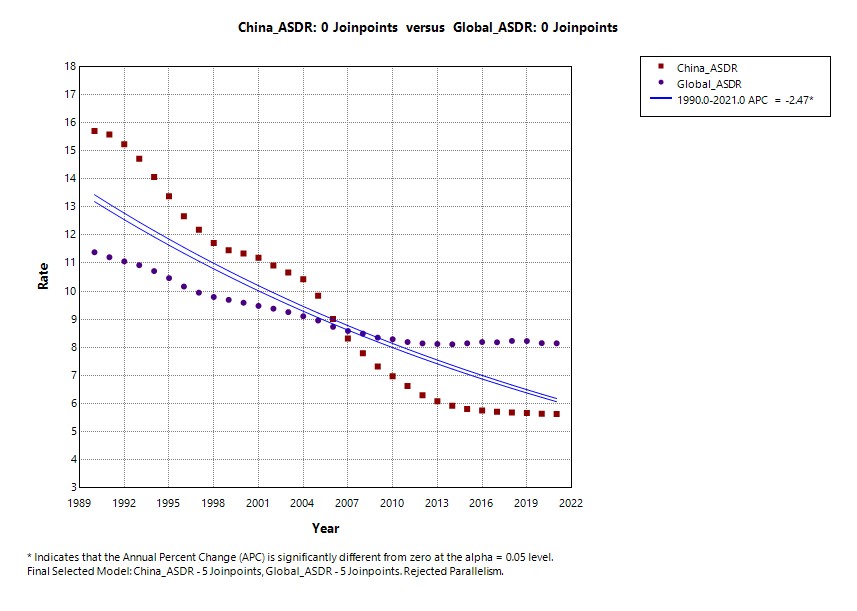


# Supplementary Table S2. Estimated Coverage Rates of the New Rural Cooperative Medical Scheme (NRCMS) in China, 2004–2010

| Year | Approximate Coverage Rate (%) | Source or Reference |
| --- | --- | --- |
| 2004 | ~21–23% | Pan B et al. Health Policy Plan. 2017;32(5):634–46. |
| 2008 | 90% | Yip WC et al. Lancet. 2012;379(9818):833–42. |
| 2010 | 96.6% | Yip WC et al. Lancet. 2012;379(9818):833–42. |

# Supplementary Table S3. Key Health Policies in China (1990–2021) and Their Potential Implications for Urolithiasis Burden

| Year | Policy Event | Potential Relevance to Urolithiasis | Source / Reference |
| --- | --- | --- | --- |
| 1994 | National Health Insurance Pilots (Zhenjiang/Jiujiang) | The launch of the Urban Employee Basic Medical Insurance (UEBMI) laid the foundation for China's health insurance system. | https://doi.org/10.1016/S0277-9536(01)00306-9 |
| 1998 | Establishment of Urban Employee Basic Medical Insurance (UEBMI) | Urban employees were the first to benefit from insurance, improving access to diagnosis and treatment. | https://en.wikipedia.org/wiki/Health_insurance_in_China |
| 2000 | Approval for Establishment of Private and Non-Profit Hospitals | Diversification of healthcare services, increasing options and resources for urolithiasis diagnosis and treatment. | https://content.sph.harvard.edu/wwwhsph/sites/1989/2020/04/NGO06.pdf |
| 2003 | Launch of New Rural Cooperative Medical Scheme (NRCMS) | Expanded insurance coverage in rural areas, enhancing access to primary care and improving urolithiasis diagnosis and intervention opportunities. | https://www.sciencedirect.com/science/article/pii/S0168851015001864 |
| 2004 | Launch of National Internet-based Infectious Disease Reporting System | Strengthening the national public health monitoring system, facilitating informatized support and early disease detection. | https://www.frontiersin.org/journals/public-health/articles/10.3389/fpubh.2021.679540/full |
| 2007 | Expansion of Urban Resident Basic Medical Insurance (URBMI) | Coverage for non-employed populations (children, elderly, etc.), improving access to care and financial protection for stone disease patients. | https://documents1.worldbank.org/curated/en/627271468010854683/pdf/584120NWP0V10P1tegratedInsurance1ES.pdf |
| 2009 | Introduction of Essential Medicines System and Drug List | Basic drugs provided in primary healthcare institutions, benefiting chronic disease management and urolithiasis prevention (e.g., antihypertensives, diuretics). | https://www.spglobal.com/marketintelligence/en/mi/country-industry-forecasting.html?ID=106595188 |
| 2009 | First-Stage Healthcare Reform: Five Major Goals | Expanded insurance coverage, improved public health services, and enhanced primary care capacity provide policy support for urolithiasis burden management. | https://jhmhp.amegroups.org/article/view/4264/5066 |
| 2010 | Pilot Reform of Public Hospitals | Improved efficiency in large hospitals' diagnostic and treatment processes may facilitate standardized care for urolithiasis patients. | https://www.frontiersin.org/journals/health-services/articles/10.3389/frhs.2023.1079370/full |
| 2012 | National Plan for NCD Prevention and Treatment (2012–2015) | Targeted control of obesity, hyperglycemia, and other risk factors to indirectly reduce the urolithiasis burden. | https://www.iccp-portal.org/system/files/plans/National%20Plan%20for%20NCD%20Prevention%20and%20Treatment%202012-2015.pdf |
| 2016 | Integration of Urban and Rural Resident Medical Insurance | Unified insurance benefits improve healthcare access for rural and peri-urban populations, promoting early diagnosis and treatment of urolithiasis. | https://en.wikipedia.org/wiki/Health_insurance_in_China |
| 2016 | Healthy China 2030 Plan Outline | Promotion of healthy lifestyle interventions (e.g., salt reduction, weight control) closely linked to metabolic stone risk reduction. | https://en.wikipedia.org/wiki/Healthcare_reform_in_China |
| 2018 | Establishment of NHSA and Launch of Centralized Drug Procurement | Reduced drug prices and improved accessibility enhance affordability of urolithiasis treatment. | https://www.frontiersin.org/journals/pharmacology/articles/10.3389/fphar.2022.923209/full |
| 2020 | Optimization of Hierarchical Diagnosis and Treatment under COVID-19 Normalization | Medical consortiums and family doctor contracting strengthen primary care, supporting early screening and referral management of urolithiasis. | https://www.gov.cn/zhengce/2020-07/31/content_5531670.htm |
| 2021 | Deepening Reform of the “Three Medical Linkages” (Insurance, Services, Drugs) | Integrated reforms in insurance payment, service pricing, and drug procurement promote standardized treatment pathways and resource allocation, improving equity in urolithiasis care. | https://www.gov.cn/zhengce/2021-07/09/content_5623741.htm |
